# Systems Biology and Atomistic Simulations Reveal Multi-Target Modulation of Alzheimer’s Disease and Type 2 Diabetes by *Caesalpinia sappan* Bioactives

**DOI:** 10.3390/ijms27125300

**Published:** 2026-06-11

**Authors:** Gracia Amadea, Kumju Youn, Mira Jun

**Affiliations:** 1 Department of Health Sciences, The Graduate School of Dong-A University, Busan 49315, Republic of Korea; 2473024@donga.ac.kr; 2 Department of Food Science and Nutrition, Dong-A University, Busan 49315, Republic of Korea; kjyoun@dau.ac.kr; 3 Center for Food & Bio Innovation, DAU G-LAMP Project Group, Dong-A University, Busan 49315, Republic of Korea

**Keywords:** Alzheimer’s diseases, type 2 diabetes mellitus, *Caesalpinia sappan*, systems biology, molecular docking, molecular dynamics, density functional theory

## Abstract

Alzheimer’s disease (AD) and type 2 diabetes mellitus (T2DM) are major global health burdens that share interconnected pathological mechanisms involving impaired insulin signaling, metabolic stress, and chronic neuroinflammation. This study applied an integrative systems biology and atomistic simulation framework to investigate bioactive compounds from *Caesalpinia sappan* L. targeting shared molecular regulators linking AD and T2DM. Network topology analysis identified four central hub genes, *STAT3*, *SRC*, *HSP90AA1*, and *TP53*, representing key regulatory nodes involved in inflammatory signaling, kinase regulation, proteostasis, and cellular stress responses. Compound-specific interaction analysis revealed distinct target preferences among phytochemical subclasses. Protosappanin B showed strong binding toward both STAT3 and HSP90α, whereas flavonols including quercetin and rhamnetin exhibited high affinity for SRC, and the chalcone derivative sappanchalcone preferentially interacted with TP53. Atomistic molecular dynamics simulations and MM-PBSA calculations supported stable protein ligand interactions and favorable binding energetics, while density functional theory analysis indicated electronic properties consistent with sustained intermolecular interactions. Collectively, these findings suggest that structurally distinct subclasses of *C. sappan* phytochemicals converge on complementary regulatory hubs within the shared AD and T2DM molecular network, supporting coordinated multi-target modulation of interconnected inflammatory, kinase signaling, proteostasis, and cellular stress pathways underlying AD–T2DM comorbidity.

## 1. Introduction

Alzheimer’s disease (AD) is a progressive neurodegenerative disorder and the leading cause of dementia worldwide, accounting for approximately 60–80% of dementia cases [1]. As the global population ages, it is projected that the number of people living with dementia will increase from approximately 57 million in 2019 to over 152 million by 2050 [2]. While amyloid-beta (Aβ) aggregation and tau hyperphosphorylation are well-established pathological hallmarks, accumulating evidence suggests that AD progresses through a complex interplay of multiple pathological processes. Protein aggregation, dysregulation of kinase signaling, chronic neuroinflammation, mitochondrial dysfunction, and impaired proteostasis are increasingly recognized as interconnected pathogenic mechanisms that drive the progression of AD [3,4]. These factors create a vicious cycle in which one pathological process exacerbates another, resulting in progressive neuronal loss.

Type 2 diabetes mellitus (T2DM) is a rapidly growing global health crisis, affecting over 400 million people worldwide [5]. T2DM is a chronic metabolic disorder primarily characterized by insulin resistance and impaired insulin secretion, resulting in disrupted insulin signaling and persistent hyperglycemia [6]. Although pancreatic β-cells initially compensate for insulin resistance by increasing insulin secretion, progressive β-cell dysfunction eventually limits this compensatory response and disrupts glucose homeostasis. Chronic metabolic imbalance associated with T2DM can lead to widespread systemic complications, including vascular dysfunction, oxidative stress, and inflammatory responses [7]. These metabolic disturbances have increasingly been implicated in pathological complications affecting multiple organs, including the central nervous system (CNS) [8].

Increasing evidence indicates that AD and T2DM share interconnected pathological mechanisms linking metabolic dysfunction with neurodegeneration, highlighting their convergence as complex multi-system disorders [9]. A meta-analysis of 17 studies involving approximately 1.7 million individuals demonstrated that individuals with diabetes had a 1.53-fold increased risk of developing AD (95% confidence interval (CI, 1.42–1.63) compared with non-diabetic individuals [10]. The brain is an insulin-responsive organ, and insulin signaling plays an essential role in neuronal metabolism, synaptic plasticity, and cognitive function [11]. Insulin resistance is recognized as a critical driver of dysregulated neuronal signaling pathways, including kinase-mediated phosphorylation, amyloid metabolism, and cellular stress responses [8]. Since insulin and Aβ are both substrates of insulin-degrading enzyme (IDE), elevated insulin levels have been shown to reduce Aβ clearance through competitive inhibition of IDE-mediated degradation [12]. Peripheral inflammatory signaling and insulin resistance associated with T2DM have been implicated in blood–brain barrier (BBB) dysfunction and glial activation, thereby facilitating the propagation of metabolic stress into the CNS [13]. Conversely, neurodegenerative processes associated with AD can further exacerbate metabolic imbalance through disruption of neuronal insulin signaling and neurovascular regulation [8,14]. These findings indicate that AD and T2DM converge on shared pathological processes including insulin resistance, inflammatory activation, oxidative stress, mitochondrial dysfunction, and vascular impairment [15]. Accordingly, integrative approaches are required to elucidate the complex molecular interactions underlying both AD and T2DM.

Systems biology provides a framework for investigating complex diseases by integrating multiple biological layers, including genes, proteins, and metabolic pathways, thereby enabling the identification of shared regulatory hubs within interconnected signaling networks [16]. In this context, natural products represent a valuable source of multi-target agents due to the structural diversity and inherent polypharmacological properties of plant-derived secondary metabolites [17,18]. However, identifying the specific active constituents responsible for these effects has traditionally required extensive experimental screening and long development timelines [19]. Recent advances in in silico systems pharmacology have facilitated the rapid prioritization and structural evaluation of natural product-derived compounds, enabling systematic exploration of their interactions with disease-associated molecular networks [20].

Among herbal plants, *Caesalpinia sappan* L. (*C. sappan*), commonly known as sappan wood, is a member of the Fabaceae family native to Southeast Asia and possesses a long history in regional ethnomedicine [21]. In particular, the heartwood has been used in traditional medicine typically as a decoction to alleviate postpartum abdominal pain, cardiovascular and gastrointestinal disorders, diabetes, infections, and inflammatory diseases [22]. Phytochemical studies have revealed that the heartwood of *C. sappan* contains structurally unique phenolic components, including brazilin-type compounds, protosappanin-type, homoisoflavonoids, chalcone derivatives, and xanthone derivatives [23,24,25]. These structurally diverse phenolic scaffolds provide chemically distinct ligands capable of interacting with multiple biological targets. Consistent with this chemical diversity, prior studies have reported that *C. sappan* extracts or single compounds exhibit biological activities associated with key processes including inflammation, oxidative stress, and metabolic dysregulation, pathophysiological mechanisms widely implicated in complex metabolic and neurodegenerative disorders [26,27].

**Figure 1:**
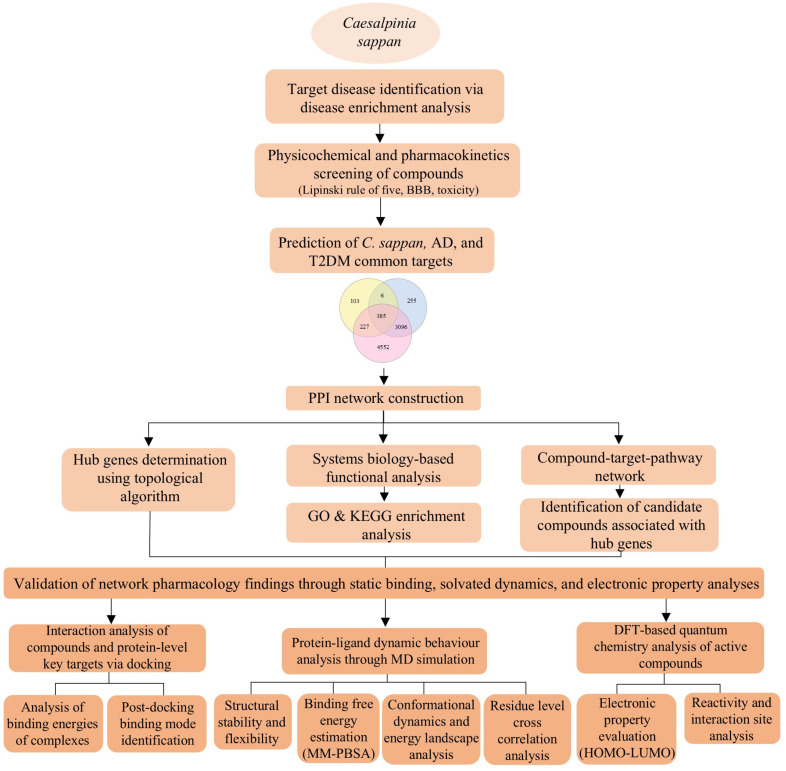
Schematic workflow of the study.

The present study systematically delineated the predicted human protein targets of *C. sappan*-derived compounds, followed by disease enrichment analysis to identify associated diseases. Shared genes associated with AD and T2DM were subsequently mapped onto the *C. sappan*-predicted target set to define common molecular targets. These targets were subjected to protein–protein interaction analysis to identify central hub genes, which were further evaluated through molecular docking, molecular dynamics simulations, binding free energy calculations, and electronic structure analysis. Importantly, this framework allows systematic evaluation of how structurally distinct subclasses of *C. sappan* phytochemicals preferentially engage different regulatory hubs within the shared AD–T2DM molecular network. This integrative strategy enables a comprehensive evaluation of whether distinct structural classes of *C. sappan* compounds interact with shared hub proteins implicated in both AD and T2DM, thereby supporting coordinated multi-target modulation within these disease-associated regulatory networks. The overall workflow of this investigation is illustrated in Figure 1.

## 2. Results

### 2.1. Prediction of C. sappan-Associated Diseases

Compounds derived from *C. sappan* were collected from the Indian Medicinal Plants, Phytochemistry and Therapeutics (IMPPAT) database to investigate their potential disease associations. After removing structurally duplicated entries, 45 compounds were retained for further analysis (Supplementary Table S1). Target prediction for these compounds yielded 721 unique target genes, which were subsequently subjected to disease enrichment analysis using the Online Mendelian Inheritance in Man (OMIM) disease gene set library.

**Figure 2:**
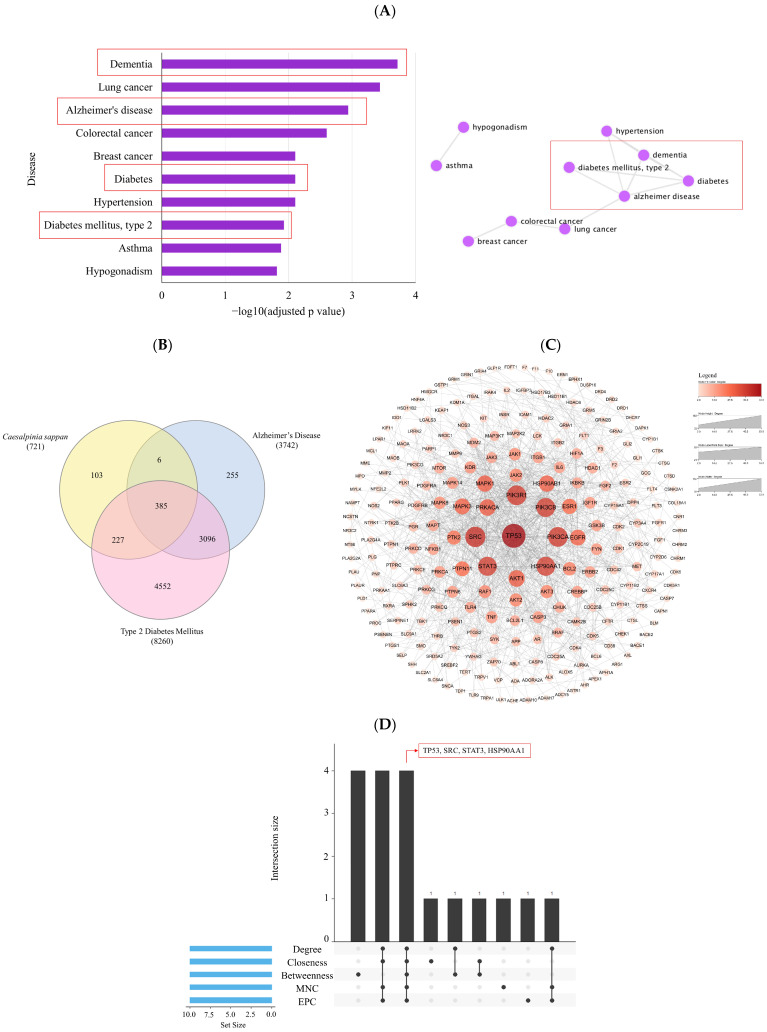
Identification and functional exploration of common targets between *C. sappan* compounds, AD, and T2DM. (**A**) Disease enrichment analysis. (**B**) Overlapping genes among predicted targets of *C. sappan* compounds, AD, and T2DM. (**C**) Protein–protein interaction (PPI) network. (**D**) The hub genes from the intersection of five topological algorithms highlighted in red box (*TP53*, *SRC*, *STAT3*, and *HSP90AA1*).

Based on adjusted *p*-value ranking, dementia and Alzheimer’s disease (AD) emerged as the most significantly enriched disease terms associated with *C. sappan*-related targets (Figure 2A). Type 2 diabetes mellitus (T2DM) was also among the top enriched disease terms and showed close network-level association with AD and dementia, suggesting potential shared molecular mechanisms.

### 2.2. Identification of AD–T2DM–Associated Targets of C. sappan Compounds

Following the identification of disease relevance linking *C. sappan*-associated targets to AD and T2DM, compounds were further prioritized to define disease-relevant molecular targets. Among the 45 non-duplicated *C. sappan* compounds, 28 satisfied the predefined physicochemical, pharmacokinetic, and safety-related criteria and were retained for subsequent target prediction and network analyses (Table 1).

Genes associated with AD and T2DM were retrieved from the GeneCards database, yielding 3742 AD-related genes and 8260 T2DM-related genes (Supplementary Table S2). Integration of the predicted targets of the selected *C. sappan* compounds with the AD- and T2DM-associated gene sets identified 385 shared genes, which were defined as common molecular targets implicated in AD–T2DM comorbidity (Figure 2B, Supplementary Table S3). These shared targets were subsequently used for PPI network construction and downstream pathway analyses.

### 2.3. Construction of the PPI Network and Identification of Hub Genes

A PPI network was constructed to systematically evaluate the interaction structure of the shared AD- and T2DM-associated targets using the STRING database and was visualized in Cytoscape (Figure 2C). The resulting network consisted of 238 nodes and 1070 edges, representing high-confidence functional associations among the target proteins. To identify key regulatory genes within the network, topological analysis was performed using five centrality parameters implemented in the CytoHubba plug-in, including degree, closeness, betweenness, maximum neighborhood component (MNC), and edge percolated component (EPC).

For each topological parameter, the top 10 ranked genes were extracted, and genes consistently identified across all five metrics were defined as hub genes to improve the reliability of hub gene identification within the interaction network (Figure 2D), consistent with the hub prioritization strategy described by Chin et al. (2014) [28]. Four genes, *STAT3*, *SRC*, *HSP90AA1*, and *TP53* were identified as hub genes, reflecting their central regulatory roles within the shared AD–T2DM interaction network.

### 2.4. Compound–Target–Pathway Network Analysis and Candidate Compound Selection

**Figure 3:**
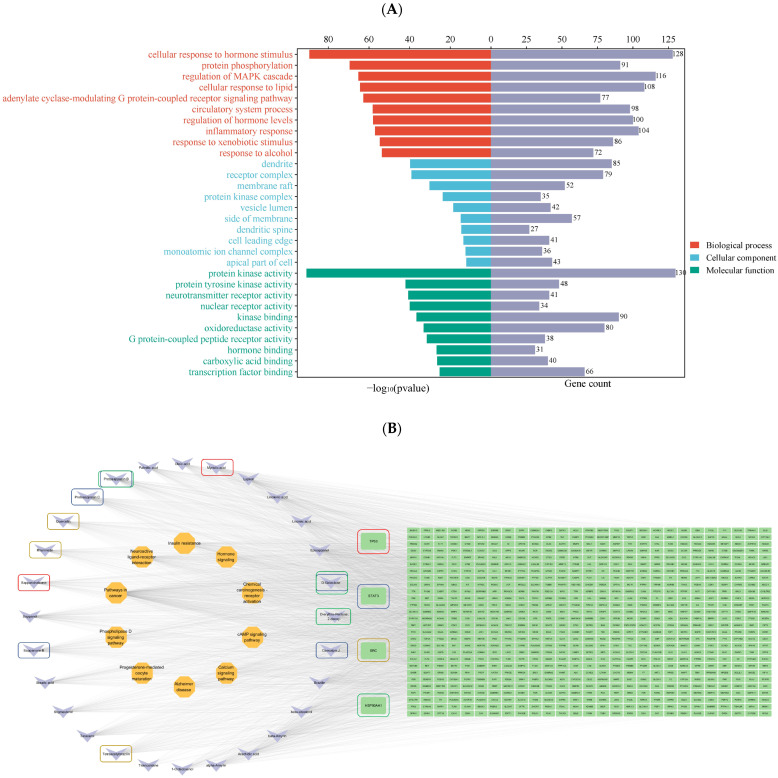
Functional characterization of shared target genes between AD, T2DM, and *C. sappan*. (**A**) Gene ontology (GO) enrichment analysis illustrating biological processes, molecular functions, and cellular components of the shared target genes. (**B**) Compounds-target-pathway network based on Kyoto Encyclopedia of Genes and Genome (KEGG) enrichment, visualizing the relationship among *C. sappan*-derived compounds, their corresponding target genes, and AD- and T2DM-related signaling pathways, with compounds and their associated hub genes highlighted in matching colors.

Gene Ontology (GO) enrichment analysis of the shared AD- and T2DM-associated targets revealed significant enrichment across biological process, cellular component, and molecular function categories (Figure 3A, Supplementary Table S4). Within the biological process category, the target genes were predominantly associated with protein phosphorylation, regulation of the MAPK cascade, cellular response to lipid, inflammatory response, and response to xenobiotic stimulus. These enrichments correspond to signal transduction, metabolic regulation, and stress-responsive processes. In the cellular component category, enriched terms included receptor complex, membrane raft, protein kinase complex, and dendrite-related structures, indicating that the target genes are primarily localized to membrane-associated and synapse-related signaling compartments. In parallel, molecular function analysis highlighted protein kinase activity, protein tyrosine kinase activity, neurotransmitter receptor activity, nuclear receptor activity, and transcription factor binding, reflecting the predominance of receptor-mediated and kinase-driven signaling pathways.

To further elucidate the relationships among compounds, targets, and pathways, a compound–target–pathway network was constructed based on Kyoto Encyclopedia of Genes and Genomes (KEGG) enrichment results. Mapping of hub genes within this network revealed distinct compound interaction profiles for each hub gene (Figure 3B). *SRC* was connected with quercetin, rhamnetin, and tetraacetylbrazilin, whereas *STAT3* exhibited interactions with protosappanin B, caesalpin J, D-galactose, protosappanin C, and sappanone B. *TP53* was associated with myristic acid and sappanchalcone, while *HSP90AA1* showed interactions with protosappanin B, 2-deoxy-D-erythro-pentose, and D-glucose. These hub gene–compound associations were distributed across KEGG pathways including Alzheimer’s disease, insulin resistance, neuroactive ligand–receptor interaction, calcium signaling pathway, and cAMP signaling pathway, as identified by pathway enrichment analysis. Collectively, these enrichments indicate that the shared targets are concentrated in kinase-driven, receptor-associated, and stress-responsive signaling modules that potentially link neurodegenerative and metabolic disease mechanisms.

### 2.5. Molecular Docking Validation of Candidate Compounds Targeting Hub Proteins

**Figure 4:**
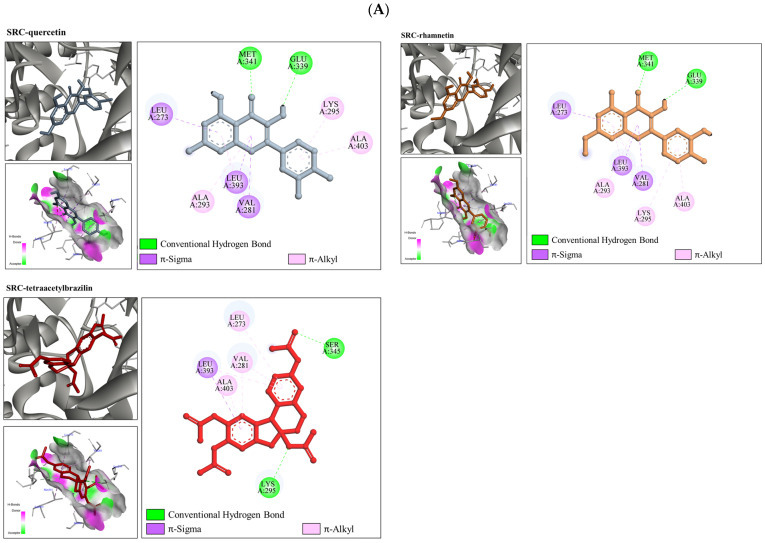

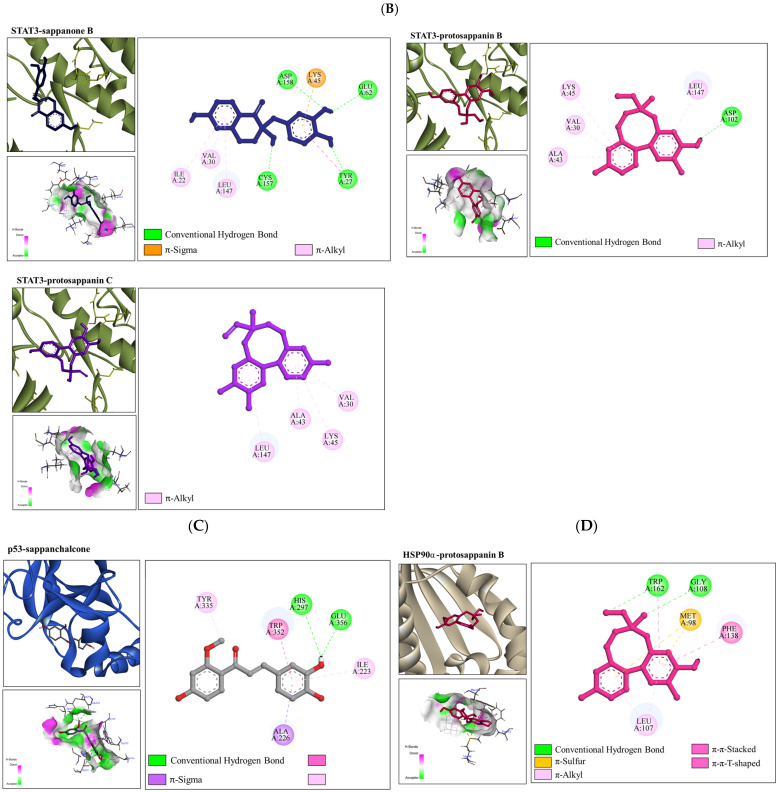
Visualization of molecular docking poses and protein–ligand interactions. Each panel shows the full protein–ligand complex structure, a focused view of the binding pocket with ligand interactions and surrounding residues, and a 2D interaction diagram illustrating specific residues and interaction types. Green and magenta surfaces indicate hydrogen bond acceptor and donor regions within the binding pocket, respectively. (**A**) SRC, (**B**) STAT3, (**C**) p53, and (**D**) HSP90α.

Based on the compound–target–pathway network analysis, representative hub gene–compound complexes were selected for subsequent molecular docking analysis to evaluate their binding feasibility and interaction characteristics at the structural level. All hub gene-associated compounds were subjected to molecular docking, and the complete binding affinities and interaction profiles are summarized in Table 2. Compounds exhibiting binding affinities of ≤−7.5 kcal/mol were selected for structural visualization, and their representative binding poses are shown in Figure 4. The docking protocol was validated by RMSD-based re-docking of native ligands into their corresponding binding sites. All systems exhibited RMSD values below 2.0 Å, confirming the reliability of the docking procedure (Supplementary Table S5).

For SRC, quercetin and rhamnetin exhibited binding affinities (−9.1 kcal/mol) comparable to that of the FDA-approved inhibitor dasatinib (−9.2 kcal/mol). Rhamnetin, a methylated derivative of quercetin, exhibited the same binding affinity (−9.1 kcal/mol) and formed hydrogen bonds with Met341 and Glu339, along with conserved hydrophobic contacts, consistent with a similar binding pose within the SRC active site. Tetraacetylbrazilin, a brazilin derivative, exhibited a strong binding affinity of −8.7 kcal/mol and engaged key π-alkyl interactions at Ala403 and Val281, residues consistently involved in high-affinity SRC–ligand complexes.

In contrast to SRC, STAT3 docking results showed distinct binding patterns among the tested compounds. Protosappanin B and protosappanin C exhibited strong binding affinities to STAT3 (−8.4 and −8.3 kcal/mol, respectively), exceeding those of the reference compound celecoxib (−8.1 kcal/mol) and the native ligand (−6.5 kcal/mol). Despite their structural similarity, protosappanin B formed a stabilizing hydrogen bond with Asp102, whereas protosappanin C achieved a comparable binding affinity predominantly through π-alkyl interactions in the absence of hydrogen bonding. Caesalpin J showed a moderate binding affinity to STAT3 (−7.1 kcal/mol) and formed hydrogen bonds with Gln96 and Asp158 (Table 2), reflecting a less favorable interaction profile compared with the protosappanin derivatives. By contrast, D-galactose exhibited a markedly weaker binding affinity (−4.7 kcal/mol) and limited interaction features within the STAT3 binding pocket.

Within the p53 binding pocket, sappanchalcone, a chalcone-type phenolic compound, adopted a binding pose closely aligned with those of the native ligand and the known p53 inhibitor, exhibiting a binding affinity of −7.8 kcal/mol. This binding affinity exceeded that of the native ligand (−7.2 kcal/mol) and was comparable to that of the known inhibitor PhiKan083 (−7.5 kcal/mol). The sappanchalcone–p53 complex was stabilized by hydrogen bonds with His297 and Glu356, together with hydrophobic interactions involving Tyr335 and Ala226, residues that were also engaged by both the inhibitor and the native ligand. By comparison, myristic acid showed a weaker binding affinity to p53 (−5.1 kcal/mol) and limited interaction features, primarily involving hydrogen bonding with Asn265 and Lys294 (Table 2).

Among the ligands docked to HSP90α, protosappanin B exhibited a strong binding affinity of −8.6 kcal/mol, exceeding that of the well-known HSP90 inhibitor geldanamycin (−8.2 kcal/mol). Importantly, protosappanin B exhibited favorable binding interactions with both STAT3 and HSP90α, highlighting its potential multi-target binding behavior. In the HSP90α complex, protosappanin B shared key hydrophobic interactions observed in the native ligand complex, including a π-alkyl interaction at Leu107 and π–π stacking with Phe138, while additionally forming hydrogen bonds with Trp162 and Gly108. Other tested saccharide-based ligands, including D-galactose and 2-deoxy-D-erythro-pentose, showed weak binding affinities (>−5.5 kcal/mol) and limited interaction features. Overall, these docking results indicate that several *C. sappan* phytochemicals exhibit favorable binding affinities toward key regulatory hub proteins associated with the AD–T2DM network.

Several key interactions identified in the present study were consistent with previously reported ligand-binding regions of the corresponding target proteins. In STAT3, protosappanin derivatives occupied the SH2 phosphotyrosine-binding pocket, a major site for STAT3 inhibitor binding [29]. In HSP90α, protosappanin B interacted with residues including Phe138 within the N-terminal ATP-binding pocket, consistent with the HSP90α –geldanamycin co-crystal structure [30]. Similarly, SRC ligands interacted with Met341 and Lys295, key residues involved in Src inhibitor binding [31]. In p53, sappanchalcone shared contact residues, including Ala226, with the native ligand and the reference ligand PhiKan083 within the Y220C ligand-binding cavity previously characterized for mutant p53 stabilizers [32]. These observations support the biological plausibility of the predicted docking poses.

### 2.6. MD-Based Evaluation of Structural Stability, Collective Motions, and Binding Energetics

Based on the molecular docking results, protein–ligand complexes exhibiting favorable binding affinities and well-defined interaction patterns were selected for subsequent molecular dynamics (MD) simulations to assess their dynamic stability and binding behavior under explicit solvent conditions. In addition to the *C. sappan*-derived compounds, well-characterized drugs reported to form stable complexes with each target protein were included in the MD simulations as benchmark systems for comparative analysis [33]. Overall, the MD simulations indicate that several *C. sappan*-derived ligands maintain stable interactions with their respective hub proteins, supporting the docking-based binding predictions.

#### 2.6.1. Dynamic Stability and Conformational Flexibility of Protein–Ligand Complexes

**Figure 5:**
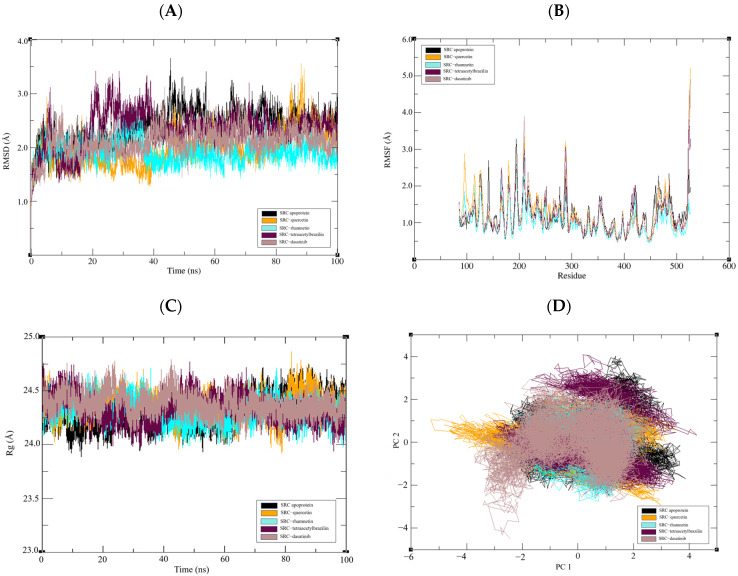
Conformational stability and dynamic adaptability of SRC–ligand complexes during MD simulations. (**A**) RMSD (**B**) RMSF (**C**) Rg (**D**) PCA.

As shown in Figure 5A, the root-mean-square deviation (RMSD) profiles of the SRC–ligand complexes indicated that all ligand-bound systems reached structural equilibrium after approximately 40 ns. The apoprotein exhibited a higher average RMSD value (2.4 Å), whereas ligand binding reduced backbone fluctuations, with average RMSD values of 2.0 Å for quercetin, 1.9 Å for rhamnetin, and 2.3 Å for tetraacetylbrazilin. Root-mean-square fluctuation (RMSF) analysis revealed largely overlapping fluctuation profiles along the SRC sequence (Figure 5B), with pronounced flexibility consistently localized at Ser209, Asn287, and the C-terminal residue Gln526, irrespective of ligand binding. Radius of gyration (Rg) analysis further showed that all SRC–ligand complexes retained compact conformations throughout the simulation (average Rg: 24.30–24.47 Å; Figure 5C). In the principal component analysis (PCA) projection, overall conformational sampling remained limited across all SRC-associated systems, with the SRC–rhamnetin complex exhibiting the most confined distribution in principal component (PC) space (Figure 5D).

**Figure 6:**
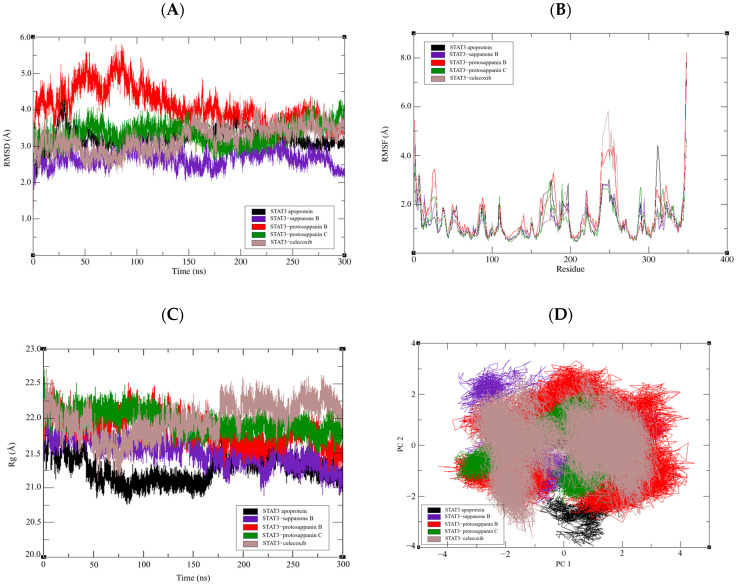
Conformational stability and dynamic adaptability of STAT3–ligand complexes during MD simulations. (**A**) RMSD (**B**) RMSF (**C**) Rg (**D**) PCA.

STAT3 complexes exhibited broader dynamic variation over 300 ns (Figure 6). The unbound protein maintained an average RMSD of 3.2 Å. Among ligands, sappanone B showed the lowest deviation (2.7 Å), while protosappanin C (3.3 Å) and celecoxib (3.2 Å) remained close to the apoprotein. In contrast, protosappanin B demonstrated the highest RMSD (4.1 Å), yielding the highest deviation among the tested complexes (Figure 6A). RMSF profiles were generally comparable; however, residue 348 consistently represented the dominant fluctuation site. The maximum RMSF reached 8.2 Å in the protosappanin B complex and 8.1 Å in the apoprotein, whereas lower peaks were observed for protosappanin C (4.7 Å) and celecoxib (5.8 Å) (Figure 6B). Although slight differences were detected in global dimensions (Rg: 21.6–22.2 Å), overall compactness was preserved throughout the trajectory (Figure 6C). PCA analysis revealed partially overlapping conformational clusters, with protosappanin B sampling a comparatively wider region of PC space (Figure 6D). Additional trajectory quality analyses of the STAT3–protosappanin B complex (Supplementary Table S6) indicated that the ligand remained associated with the binding pocket throughout the simulation, with mean binding-site Cα RMSD and ligand RMSD values of 3.2 and 3.5 Å, respectively. Cluster analysis identified a dominant binding mode occupying 56.0% of the trajectory, whereas the two most populated clusters together accounted for 86.7% of sampled conformations, suggesting that the elevated global RMSD primarily reflected protein conformational adaptation rather than ligand dissociation.

**Figure 7:**
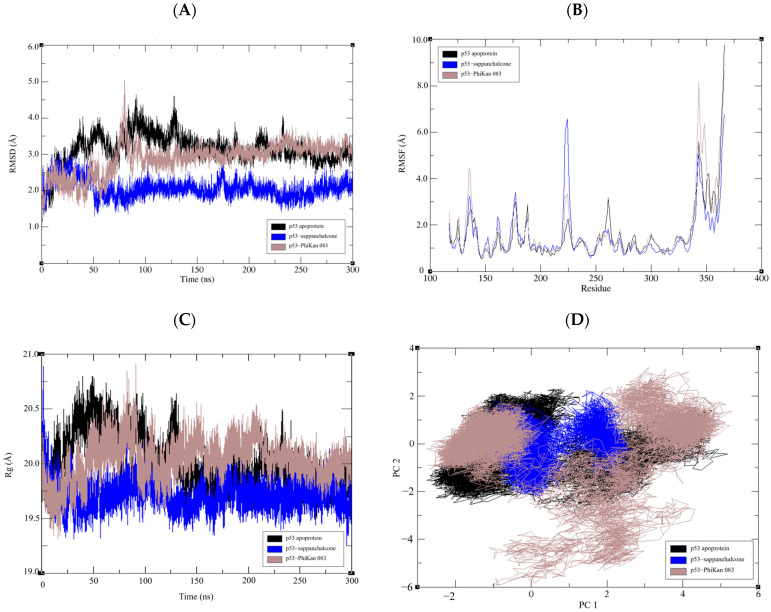
Conformational stability and dynamic adaptability of p53–ligand complexes during MD simulations. (**A**) RMSD (**B**) RMSF (**C**) Rg (**D**) PCA.

For p53, ligand-dependent differences were more clearly reflected in backbone deviation (Figure 7A). The apoprotein showed an average RMSD of 3.1 Å, whereas sappanchalcone reduced this value to 2.1 Å. PhiKan083 yielded an intermediate deviation of 2.9 Å. Residue-level analysis identified residue 366 as the principal contributor to flexibility, reaching 9.0 Å in the apoprotein. This peak decreased to 5.7 Å in the sappanchalcone-bound system and to 7.7 Å in the PhiKan083 complex (Figure 7B). Apart from this region, fluctuation profiles remained largely similar. The radius of gyration varied minimally among systems (22.0–22.1 Å), indicating maintenance of overall fold integrity (Figure 7C). In PCA space, the sappanchalcone complex formed a more concentrated cluster relative to the broader distributions observed for the apoprotein and reference inhibitor (Figure 7D).

**Figure 8:**
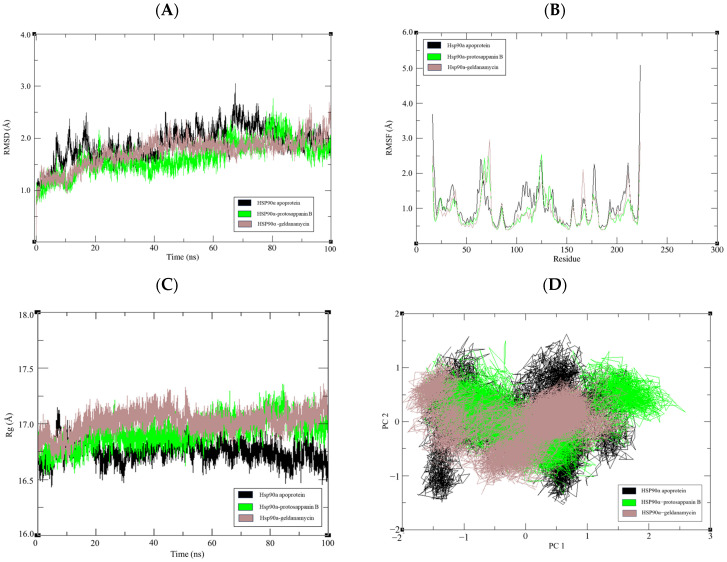
Conformational stability and dynamic adaptability of HSP90α–ligand complexes during MD simulations. (**A**) RMSD (**B**) RMSF (**C**) Rg (**D**) PCA.

In the HSP90α systems, structural deviations were comparatively small across all trajectories (Figure 8A). The apoprotein exhibited an average RMSD of 1.9 Å, while protosappanin B showed a slightly lower value (1.6 Å), comparable to geldanamycin (1.7 Å). RMSF values were generally modest, with maximal fluctuations observed at residue 223 in both the apoprotein (5.1 Å) and protosappanin B complex (2.9 Å), whereas geldanamycin displayed a distinct peak at residue 73 (3.0 Å) (Figure 8B). The compact nature of HSP90α was maintained throughout the simulation (Rg: 16.7–17.0 Å; Figure 8C). PCA projections revealed overlapping conformational regions among systems, without pronounced dispersion (Figure 8D). These results indicate that several *C. sappan*-derived ligands maintain stable interactions with their respective hub proteins during the simulations, supporting the docking-based binding predictions.

#### 2.6.2. Binding Free Energy Profiles of Protein–Ligand Complexes Assessed by MM-PBSA

Binding free energy calculations using the Molecular Mechanics Poisson–Boltzmann Surface Area (MM-PBSA) approach were conducted to quantitatively assess ligand–protein interaction strengths, as widely applied in recent MD-based binding studies [34]. The calculated energy components and total binding energies are summarized in Table 3.

For SRC, quercetin (−26.72 ± 5.23 kcal/mol) and tetraacetylbrazilin (−22.46 ± 3.79 kcal/mol) exhibited binding free energies comparable to or lower than that of the SRC–dasatinib reference complex (−23.88 ± 3.85 kcal/mol), whereas rhamnetin showed a relatively less favorable but still appreciable binding affinity (−17.66 ± 6.47 kcal/mol). Notably, quercetin displayed a slightly more favorable total binding free energy than dasatinib, indicating binding strength comparable to the approved inhibitor. These trends were primarily driven by favorable van der Waals contributions, partially offset by polar solvation penalties. The STAT3 complexes displayed a wider dispersion of total binding energies. Protosappanin B produced the lowest total value (−32.15 ± 4.92 kcal/mol), while sappanone B (−27.47 ± 7.11 kcal/mol) and protosappanin C (−27.45 ± 2.97 kcal/mol) exhibited similar energetic magnitudes. In contrast, the STAT3–celecoxib complex showed a near-neutral total binding energy (0.90 ± 3.28 kcal/mol). All *C. sappan*-derived ligands demonstrated more favorable total binding energies than celecoxib, indicating stronger predicted binding stability relative to the reference drug. Notably, the three *C. sappan*-derived ligands consistently maintained negative GGAS values (−63.14 to −74.81 kcal/mol), which were partially counterbalanced by solvation terms, yielding overall favorable total energies.

In the p53 system, the total binding free energy of sappanchalcone was calculated as −24.09 ± 3.94 kcal/mol, slightly lower than that of the reference inhibitor PhiKan083 (−21.72 ± 2.69 kcal/mol). This indicates that sappanchalcone achieved a binding affinity comparable to, and marginally more favorable than, the established p53 stabilizer. The PhiKan083 complex exhibited an exceptionally large electrostatic contribution (−333.29 ± 18.86 kcal/mol), which was nearly compensated by an equally high polar solvation term (340.41 ± 18.32 kcal/mol), resulting in a moderate net binding energy. In contrast, sappanchalcone showed smaller but more proportionate gas-phase and solvation components, leading to a comparable overall binding magnitude without extreme compensation effects.

For HSP90α, protosappanin B displayed a total binding free energy of −18.55 ± 3.92 kcal/mol, whereas geldanamycin showed a lower value (−25.24 ± 3.34 kcal/mol). Both complexes were primarily stabilized by favorable van der Waals interactions (−36.77 ± 0.76 and −39.41 ± 2.84 kcal/mol, respectively), with electrostatic contributions remaining modest. Although protosappanin B exhibited a less negative total binding free energy than geldanamycin, it maintained a negative binding energy and also showed favorable binding energetics toward STAT3. These MM-PBSA calculations further support the binding tendencies observed in the docking and MD analyses, highlighting protosappanin B, quercetin, sappanchalcone, and tetraacetylbrazilin as energetically favorable binders across multiple hub proteins.

#### 2.6.3. Free Energy Landscape (FELs) and Residue-Level Dynamic Correlations

**Figure 9:**
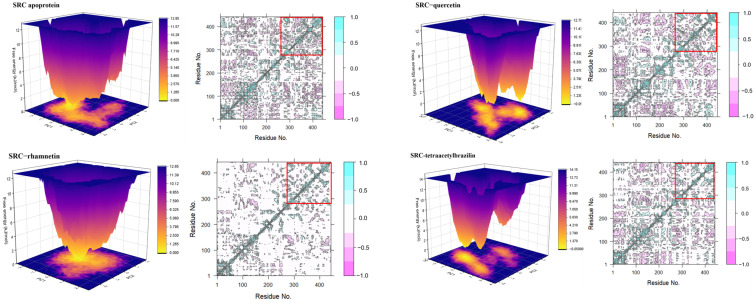

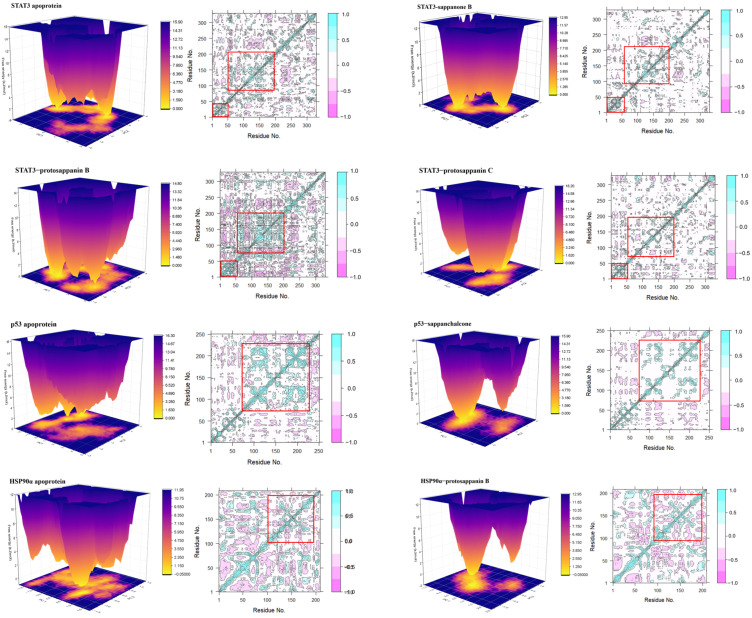
FEL 3D plot (**left**) and DCCM map (**right**) depicting conformational stability and residue level correlation of apoproteins and ligand-bound complexes. Red boxes in the DCCM map indicate regions corresponding to the binding site residues involved in protein–ligand interactions.

Free energy landscapes (FELs) and residue-level dynamic cross-correlation patterns were analyzed for all apoproteins and ligand-bound complexes. FEL representations derived from the first two principal components (PC1 and PC2) and the corresponding dynamic cross-correlation matrix (DCCM) maps are shown in Figure 9.

Among the SRC-associated systems, the SRC–rhamnetin complex exhibited a single, well-defined deep energy basin with limited conformational dispersion. This behavior was accompanied by reduced anti-correlated motions in the corresponding DCCM maps, particularly within residues 300–400. Relative to the apoprotein, the rhamnetin complex showed attenuated anti-correlated fluctuations in this region, suggesting enhanced residue-level coordination upon ligand binding.

STAT3 complexes occupied broader conformational spaces overall; however, the STAT3–protosappanin B system displayed clearly defined low-energy minima. Quantitative FEL analysis showed that the STAT3–protosappanin B complex exhibited a single dominant energy basin without an apparent energy barrier between minima and the largest sampled basin area among the STAT3 systems (30.0155 nm^2^; Supplementary Table S7), indicating broad but continuous conformational sampling. DCCM analysis further revealed enhanced correlated motions among residues within the binding-site region. In particular, the maximum correlation coefficient increased from 0.9489 in the STAT3 apoprotein to 0.9581 in the STAT3–protosappanin B complex, while the number of strongly correlated residue pairs (Cij > 0.5) increased from 1790 to 2955 pairs (Supplementary Table S8). The protosappanin B complex showed strengthened positive correlations relative to the apoprotein, particularly in the highlighted residue cluster, indicating coordinated intramolecular dynamics.

In the p53 system, the sappanchalcone-bound complex sampled fewer and more concentrated energy basins than the apoprotein. Relative to the apoprotein, sappanchalcone exhibited a similarly stable but more centrally localized low-energy minimum, indicating comparable conformational confinement. DCCM analysis showed strengthened inter-residue correlations within residues 100–250 relative to the apoprotein, indicating coordinated intramolecular dynamics within the highlighted residue cluster.

For HSP90α, binding of protosappanin B resulted in a distinct low-energy basin in the FEL. The protosappanin B complex maintained a similarly compact and well-defined energy basin, indicating preserved structural stability. In the DCCM maps, protosappanin B binding was associated with a reduction in anti-correlated motions within the highlighted region, indicating coordinated and dynamically stable protein–ligand interactions. These FEL and DCCM analyses further indicate that several *C. sappan*-derived ligands stabilized conformational states and promote coordinated residue-level dynamics within the hub proteins.

### 2.7. DFT-Based Quantum Chemistry Analysis of C. sappan Active Compounds

To elucidate the electronic features underlying the reactivity of the validated *C. sappan* active compounds, frontier molecular orbital (FMO) parameters were evaluated, and the results are summarized in Table 4. The HOMO–LUMO energy gap (ΔE) reflects the energetic feasibility of electron transfer, with the HOMO associated with electron-donating capacity and the LUMO with electron-accepting capacity.

**Figure 10:**
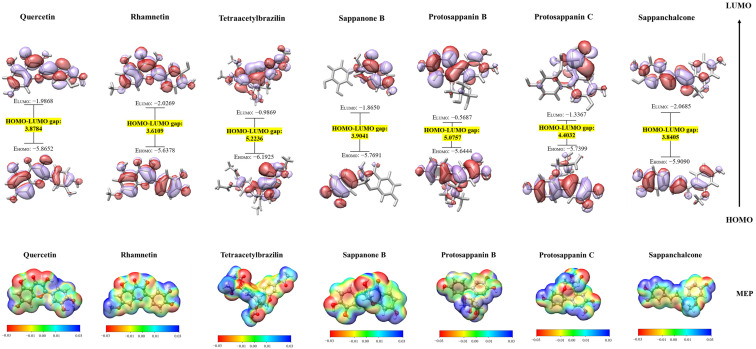
Electronic structure and electrostatic properties of *C. sappan* active compounds.

Among the seven compounds, rhamnetin exhibited the smallest HOMO-LUMO gap value (3.6109 eV), followed by sappanchalcone (3.8405 eV), quercetin (3.8784 eV), and sappanone B (3.9041 eV), indicating relatively higher electronic softness and greater chemical reactivity. In contrast, protosappanin C (4.4032 eV), protosappanin B (5.0757 eV), and tetraacetylbrazilin (5.2236 eV) displayed progressively larger energy gaps, suggesting lower electronic reactivity and reduced molecular softness (Figure 10).

The corresponding HOMO and LUMO isosurfaces revealed compound-specific orbital localization patterns, with electron density primarily distributed over phenolic and conjugated ring systems, consistent with their involvement in intermolecular interactions. To further characterize surface charge properties, molecular electrostatic potential (MEP) maps were generated (Figure 10). The MEP surfaces revealed distinct regions of negative electrostatic potential localized around oxygen-containing functional groups, including hydroxyl and carbonyl moieties, whereas positively charged regions were mainly distributed over aliphatic or less substituted aromatic areas. These electrostatic features may contribute to hydrogen bonding and electrostatic contacts observed in the molecular docking and MD simulations. Moreover, Fukui function analysis was performed to further evaluate potential reactive sites of the selected compounds (Supplementary Table S9). Atoms with relatively higher electrophilic $\left(f^{-}\right)$ and nucleophilic $\left(f^{+}\right)$ indices were mainly distributed around oxygen-containing functional groups and conjugated aromatic regions, consistent with the hydrogen-bonding and π-mediated interaction patterns observed in the docking analyses. These findings further support the contribution of localized electronic reactivity to the observed protein–ligand interaction profiles. Together, these density functional theory (DFT)-derived electronic properties provide complementary information related to the interaction characteristics of *C. sappan* bioactives with the identified hub proteins.

## 3. Discussion

AD and T2DM share overlapping molecular mechanisms characterized by chronic inflammation, dysregulated phosphorylation signaling, impaired proteostasis, and oxidative metabolic stress [14]. In the present study, analysis of the *C. sappan*-derived target network within the AD–T2DM shared gene set highlighted *STAT3*, *SRC*, *HSP90AA1*, and *TP53* as hub genes. These hub genes represent complementary regulatory layers encompassing inflammatory transcription (*STAT3*), receptor-associated kinase signaling (*SRC*), molecular chaperone-mediated proteostasis (*HSP90AA1*), and stress-responsive checkpoint control (*TP53*) [35,36,37,38]. Although these regulatory processes are often examined independently, accumulating evidence indicates substantial molecular convergence between neurodegeneration and metabolic dysfunction [8,39].

The structural diversity of *C. sappan*-derived compounds was reflected in their differential associations with the identified hub genes in AD–T2DM. A major finding of the present study is that structurally distinct subclasses of *C. sappan* phytochemicals preferentially engage different regulatory hubs within the shared AD–T2DM molecular network. Protosappanin B and protosappanin C, belonging to a characteristic protosappanin subclass of *C. sappan*, were primarily linked to STAT3, a transcription factor that drives pro-inflammatory cytokine expression and contributes to insulin resistance and glial activation in AD and T2DM [38,40]. Protosappanin B was additionally associated with HSP90AA1, which encodes the molecular chaperone HSP90α involved in tau stabilization and maintenance of proteostasis under metabolic stress conditions [41,42]. Homoisoflavonoid constituents such as sappanone B were also connected to STAT3, indicating that multiple structurally distinct subclasses within *C. sappan* converge on STAT3-mediated regulatory pathways. The chalcone-type compound sappanchalcone corresponded to TP53, whose encoded protein p53 regulates mitochondrial stress responses and apoptosis triggered by oxidative and metabolic imbalance [43,44]. In contrast, flavonols including quercetin and rhamnetin, together with the brazilin-derived tetraacetylbrazilin, were preferentially associated with SRC, a Src family kinase that in AD participates in Fyn-associated amyloid-β-dependent synaptotoxic signaling, and in metabolic tissues is involved in Src–JNK–mediated attenuation of insulin signaling and β-cell secretory regulation [45,46]. 

MD analyses of the STAT3 complexes revealed that protosappanin derivatives formed relatively stable interaction profiles among the evaluated compounds. Protosappanin B and protosappanin C showed binding affinities of −8.4 and −8.3 kcal/mol, respectively, exceeding that of the reference inhibitor. Their binding orientations were generally consistent with the docking poses within the STAT3 binding pocket. Although the STAT3–protosappanin B complex exhibited higher backbone RMSD values compared with the apo structure, the ligand remained associated with the STAT3 binding pocket throughout the simulation trajectory. DCCM analysis suggested coordinated residue motions surrounding the ligand-binding region, suggesting maintenance of local interaction networks rather than transient surface contacts. MM-PBSA calculations further supported sustained binding energetics under solvated conditions, indicating that the protosappanin derivatives can effectively maintain protein–ligand interactions within the STAT3 binding environment. Previous studies have reported that protosappanin-type compounds suppress JAK2/STAT3 phosphorylation in inflammatory models [47], supporting their potential association with STAT3-related signaling modulation.

Sappanone B also demonstrated stable binding to the STAT3 pocket. The STAT3–sappanone B complex exhibited the lowest backbone deviation among the tested ligands, maintaining an average RMSD of approximately 2.7 Å, which was lower than both protosappanin derivatives and the celecoxib reference drug. Unlike the protosappanin derivatives, which showed relatively confined conformational sampling, the STAT3–sappanone B complex explored a slightly broader conformational space in PCA analysis while maintaining overall structural stability. MM-PBSA calculations further supported favorable binding energetics, yielding a binding free energy of −27.47 ± 7.11 kcal/mol, comparable to that of protosappanin C. Suppression of the JAK2/STAT3 signaling pathway has also been reported in inflammatory models [48], consistent with the STAT3-associated interaction patterns observed for sappanone B in the present study.

Protosappanin B interacted with both STAT3 and HSP90α, suggesting a dual-target interaction profile not observed for other compound subclasses. Within the HSP90α complex, stabilization occurred primarily around the ATP-binding region, where reduced backbone fluctuations were observed relative to both the apo and geldanamycin-bound systems. DCCM analysis revealed attenuation of anti-correlated motions within the ATP-binding region, indicating coordinated chaperone-domain dynamics. These observations suggest that protosappanin derivatives may influence multiple regulatory layers linking inflammatory signaling and proteostasis. Structural differences between the protosappanin derivatives and sappanone B may partly explain their distinct dynamic behaviors. The multi-ring phenolic architecture of the protosappanin derivatives provides multiple potential interaction sites within the STAT3 pocket, enabling diverse residue contacts and broader conformational sampling, whereas the more compact homoisoflavonoid molecular structure of sappanone B favors a relatively restrained binding orientation.

Flavonol-associated complexes with SRC exhibited confined conformational behavior within the kinase domain. The confined conformational sampling observed in PCA space and stable radius of gyration values indicated preservation of kinase-domain compactness following ligand binding. Quercetin and rhamnetin showed high binding affinities (−9.1 kcal/mol) comparable to dasatinib and maintained lower backbone deviations relative to the apoprotein during MD simulations. MM-PBSA profiles further supported favorable binding energetics comparable to the reference inhibitor. In addition to flavonols, the brazilin-derived compound tetraacetylbrazilin also demonstrated stable association with the SRC catalytic pocket. MD simulations showed that the SRC–tetraacetylbrazilin complex maintained a relatively stable trajectory with reduced backbone deviation compared with the apoprotein, while preserving overall structural compactness throughout the simulation. Consistent with these observations, MM-PBSA analysis yielded a favorable binding free energy (−22.46 ± 3.79 kcal/mol), comparable to the SRC–dasatinib reference complex. Quercetin has also been reported to modulate Src family kinase signaling and attenuate Aβ-associated neurotoxicity [49,50], providing biological support for the potential involvement of SRC-mediated signaling pathway in AD-related pathological processes.

Sappanchalcone displayed a distinct interaction mode within the TP53 system characterized by localized conformational flexibility rather than rigid domain stabilization. This flexibility is consistent with the chalcone structure, which contains an α,β-unsaturated carbonyl linker connecting two aromatic rings. Previous studies have shown that sappanchalcone can influence p53-associated apoptotic signaling. In human oral cancer cells, sappanchalcone increased p53 expression, promoted Bax upregulation and Bcl-2 downregulation, and triggered mitochondrial apoptosis accompanied by caspase activation [51]. Similarly, sappanchalcone was reported to induce both caspase-dependent and apoptosis-inducing factor-mediated cell death in colon cancer cells, further supporting its involvement in mitochondrial stress signaling pathways [52]. Although these studies were conducted primarily in cancer models, they indicate that sappanchalcone can influence p53-associated cellular stress responses, providing functional context for the TP53 interaction observed in the present simulations.

The multi-ring bridged protosappanin scaffold, the more compact homoisoflavonoid framework of sappanone B, the planar polyphenolic architecture of flavonols, and the flexible α,β-unsaturated chalcone backbone of sappanchalcone corresponded to distinct binding modes and dynamic behaviors at STAT3, SRC, HSP90α, and TP53, indicating a clear structure–dynamics–function relationship across the *C. sappan* chemotypes. The rigid polycyclic architecture of protosappanin derivatives provides multiple spatially distributed interaction points that favor engagement with multiple residue-contact regions within the STAT3 and HSP90α binding pockets. In contrast, the comparatively planar flavonol scaffold is well-suited to the elongated SRC kinase-binding pocket, facilitating simultaneous hydrogen-bonding and hydrophobic interactions while maintaining restricted conformational mobility. The flexible chalcone linker of sappanchalcone permits adaptive conformational adjustment within the TP53 binding region, consistent with the localized flexibility observed during MD simulations. The more compact homoisoflavonoid framework of sappanone B favors a relatively restrained binding orientation within STAT3, consistent with its lower backbone deviation and confined conformational sampling during MD simulations.

Electronic structure analysis provided supplementary physicochemical information related to these interactions. Compounds such as rhamnetin, sappanone B, and sappanchalcone exhibited relatively small HOMO–LUMO gaps and polarized electrostatic surfaces, reflecting relatively higher electronic softness and distinct charge distributions. These electronic features were observed alongside hydrogen bonding and π-mediated interactions observed in the docking analysis. These electronic features provide favorable environments for interactions with amino acid residues within protein binding pockets. Although protosappanin derivatives showed moderately larger orbital gaps, their preserved steric complementarity and stable binding configurations observed during MD simulations indicate that molecular geometry and electronic distribution may contribute to their interaction characteristics with the target proteins. Overall, the DFT-derived electronic properties served primarily as complementary physicochemical descriptors supporting the structural interpretation of compound–protein interactions.

Importantly, the biological roles of STAT3 and TP53 differ across tissues and pathological contexts associated with AD and T2DM. In T2DM, persistent STAT3 activation under chronic metabolic stress has been linked to insulin resistance through dysregulated insulin signaling, whereas in AD, dysregulated STAT3 signaling contributes to neuroinflammation, glial activation, and Aβ-associated neuroinflammation and tau-related pathology [53,54,55]. Similarly, TP53 activation in T2DM is associated with systemic oxidative and metabolic stress responses, while in AD, increased TP53 activity has been implicated in neuronal apoptosis and neurodegenerative progression [44,56,57]. In particular, STAT3 activity is closely associated with phosphorylation-dependent activation, whereas TP53-mediated responses are more strongly linked to transcriptional regulation of stress- and apoptosis-related genes. Accordingly, additional studies in AD–T2DM-relevant experimental models will be important for gaining a deeper understanding of how these compound–target interactions influence downstream signaling activity and for evaluating pharmacokinetic properties, including metabolic stability, tissue exposure, and brain exposure following BBB penetration in vivo.

Collectively, these integrated findings indicate that structurally diverse constituents of *C. sappan* interact with multiple regulatory proteins involved in inflammation, kinase signaling, proteostasis, and cellular stress responses. Different structural subclasses of *C. sappan* compounds appear to engage distinct regulatory nodes within the shared molecular network linking AD and T2DM. Protosappanin derivatives were preferentially associated with STAT3 and HSP90α, flavonols and brazilin-derived compounds with SRC, and the chalcone derivative with TP53. This distribution suggests that the phytochemical diversity of *C. sappan* may enable simultaneous modulation of multiple interconnected signaling pathways linking neurodegeneration and metabolic dysfunction.

## 4. Materials and Methods

### 4.1. Collection of C. sappan Compounds and Targets

Compounds from *C. sappan* were retrieved from the IMPPAT 2.0 database (https://cb.imsc.res.in/imppat/; accessed on 8 April 2025) [58] and their SMILES structures were obtained from the PubChem database (https://pubchem.ncbi.nlm.nih.gov/; accessed on 10 April 2025). Subsequently, compound-related targets were predicted using SwissTargetPrediction (http://www.swisstargetprediction.ch/; probability > 0; accessed on 14 April 2025) and Super-PRED (https://bio.tools/superpred probability ≥ 50%; accessed on 16 April 2025) [59].

### 4.2. Prediction of Compound Target-Associated Diseases and Screening of Physicochemical and Drug-Likeness Properties

The predicted targets were analyzed using disease enrichment analysis to identify associated disease terms. The analysis was performed using the OMIM disease database in the EnrichR platform (https://maayanlab.cloud/Enrichr; accessed on 17 April 2025) and the top 10 enriched disease terms were ranked by *p*-value [60]. Then the physicochemical, toxicity, and drug-likeness properties of the compounds were evaluated using SwissADME (http://www.swissadme.ch/; accessed on 18 April 2025) [61], pkCSM (https://biosig.lab.uq.edu.au/pkcsm/; accessed on 18 April 2025) [62], and ADMETlab 2.0 (https://admetlab3.scbdd.com/; accessed on 18 April 2025) tools [63]. Key screening parameters included Lipinski’s Rule of Five (≤1 violation), topological polar surface area (TPSA), BBB permeability, AMES toxicity, and hERG inhibition risk. Compounds were retained only when they satisfied the predefined physicochemical, pharmacokinetic, and safety-related criteria across the integrated ADMET screening workflow.

### 4.3. Identification of Shared AD–T2DM-Associated Compound Targets

To identify genes associated with AD and T2DM, gene data were collected from GeneCards (https://www.genecards.org; relevance score ≥ 10; accessed on 23 April 2025) [64]. Duplicate genes were removed within the dataset and the overlapping targets between the compound and AD/T2DM-associated genes were identified using InteractiVenn (https://www.interactivenn.net/; accessed on 26 April 2025) [65]. These intersecting targets were selected for downstream protein interaction and network analyses.

### 4.4. Protein–Protein Interaction (PPI) Network Analysis and Hub Genes Prioritization

To explore functional interactions among the common target proteins, a protein–protein interaction (PPI) network was constructed using the STRING database version 12.0 (https://string-db.org/; accessed on 29 April 2025) [66]. The organism was set to *Homo sapiens*, and the minimum required interaction score was set to 0.900 (highest confidence threshold) to ensure robust interaction data. The resulting PPI network was visualized and analyzed in Cytoscape software (version 3.10.3) [67]. The CytoHubba plug-in in Cytoscape was utilized to evaluate topological features of the PPI network based on five centrality algorithms, including degree, closeness, betweenness, MNC, and EPC. The top ten genes from each centrality metric were extracted, and the UpSetR package (v1.4.0) in R (v4.2.2) was applied to determine overlapping genes across all five metrics. Genes consistently identified across all centrality algorithms were defined as hub genes. This multi-algorithm consensus strategy was used to reduce method-specific bias in hub gene prioritization.

### 4.5. Compound-Target Pathway Network Construction and Candidate Compound Selection

To investigate the functional relevance of the identified target genes, GO and KEGG enrichment analyses were carried out using the Metascape platform (https://metascape.org/; accessed on 2 May 2025) for *Homo sapiens* [68]. Multiple testing correction was performed using the Benjamini–Hochberg false discovery rate (FDR), and terms with FDR < 0.05 were considered statistically significant. GO analysis was used to classify genes into three major categories: biological process, molecular function, and cellular component, providing insight into the biological roles and molecular activities associated with the gene set. The top 10 most significantly enriched terms were plotted using https://bioinformatics.com.cn/ (accessed on 3 May 2025) tool [69]. KEGG pathway analysis was performed to identify key signaling pathways potentially involved in the biological mechanisms relevant to AD-T2DM and the compound-target network was constructed and visualized using Cytoscape. Compounds showing direct interaction with the identified hub genes were selected as candidate compounds for further validation.

### 4.6. Molecular Docking Analysis of Hub Protein–Compound Interactions

The 3D ligand structures of candidate compounds of *C. sappan* were obtained from the PubChem database in SDF format. These structures were subjected to energy minimization using UCSF Chimera version 1.19 to ensure optimal conformations for docking [70]. The minimized structures were then converted to PDBQT format using AutoDockTools version 1.5.7. Protein structures were retrieved from the Protein Data Bank (PDB), and docking preparations, including removal of water molecules and addition of hydrogen atoms, Kollman charges, and Gasteiger charges were performed in AutoDockTools [71]. Only the protein chain containing the co-crystallized ligand and corresponding binding site was retained for docking analyses, whereas additional protein chains and non-essential heteroatoms were removed during receptor preparation. Protein structures were selected from the Protein Data Bank based on crystal structure quality, resolution, and completeness of the binding-site region. Preference was given to structures with fully resolved binding-site residues and clearly defined binding pockets suitable for molecular docking. As the binding-site residues of the selected crystal structures were fully resolved, no additional loop modeling was required.

Molecular docking was performed using AutoDock Vina v1.1.2 with an exhaustiveness value of 8 [72]. All docking calculations were performed as non-covalent, site-directed rigid-receptor docking, while ligand flexibility was retained during conformational sampling. Celecoxib was used as a reference compound for STAT3 based on previous reports describing inhibitory effects on STAT3 signaling [73]. The proteins used for docking were HSP90α (PDB ID: 7LT0, resolution: 1.70 Å), SRC (PDB ID: 2H8H, resolution: 2.20 Å), STAT3 (PDB ID: 5AX3, resolution: 2.98 Å), and P53 (PDB ID: 1XQH, resolution: 1.75 Å). The selected SRC crystal structure (PDB ID: 2H8H) corresponds to the active DFG-in kinase conformation. For HSP90α, the docking grid box was centered at coordinates −32.41 Å (x), −10.91 Å (y), and −24.92 Å (z), with a box size of 20 × 20 × 20 Å. In the case of SRC, the grid box center was positioned at 16.53 Å (x), 20.96 Å (y), and 57.98 Å (z), with a box size of 25 × 25 × 25 Å. For STAT3, docking was performed using a grid centered at 15.4788 Å (x), −5.4282 Å (y), and −15.5761 Å (z), with a box size of 20 × 20 × 20 Å. Lastly, for p53, the grid box center was set at 8.48 Å (x), −15.55 Å (y), and 11.83 Å (z), with a box size of 20 × 20 × 20 Å [74].

The docking results were further analyzed by visual inspection of protein–ligand interactions using BIOVIA Discovery Studio Visualizer v25.1.0.24284. RMSD-based re-docking of native ligands was performed to validate the docking protocol, and RMSD values below 2.0 Å were considered indicative of reliable docking performance [75].

### 4.7. MD Simulation

MD simulation was performed using the GROningen MAchine for Chemical Simulations (GROMACS) 2023.3 software package [76]. The CHARMM27 force field was applied to generate topology files and define the atomic parameters for the proteins, and the TIP3P water model was used to simulate the solvent environment. Long-range electrostatic interactions were treated using the Particle Mesh Ewald (PME) method with a cutoff distance of 1.2 nm for both electrostatic and van der Waals interactions. Ligand topology parameters were generated using the CGenFF program compatible with the CHARMM27 force field. Both protein–ligand complex and apo-protein were solvated in a dodecahedral box filled with water molecules, and electrically neutralized by adding Na^+^ and Cl^−^ ions to achieve a physiological salt concentration of 0.15 M. Prior to the production run, energy minimization was conducted using the steepest descent algorithm for 50,000 steps to eliminate steric clashes and ensure a stable initial conformation. This was followed by two equilibration phases, each lasting 100 picoseconds (ps). The first phase was conducted under the NVT ensemble to stabilize the temperature at 300 K using the V-rescale thermostat, while the second phase used the NPT (isothermal–isobaric) ensemble with the Parrinello–Rahman barostat to equilibrate pressure at 1 bar. After equilibration, a 100-nanosecond (ns) production MD simulation was performed for each system using a 2 fs integration time step. For systems that did not reach a stable plateau in RMSD within 100 ns, simulations were extended to 300 ns. Trajectory coordinates were saved every 10 ps for subsequent structural and energetic analyses Supplementary Table S10 [77].

### 4.8. Post-MD Simulation Analysis of Protein–Ligand Complexes

#### 4.8.1. Structure Stability and Flexibility Analysis

Post-MD trajectory analyses were conducted to evaluate structural stability, conformational flexibility, and intermolecular interaction characteristics. The RMSD was calculated to assess the structural stability of each system by measuring the average deviation in atomic positions from the reference structure over time [78]. To evaluate local flexibility, the RMSF of individual residues was analyzed. Furthermore, the Rg was calculated to assess the degree of compactness and structural integrity of the complexes. The PCA was employed to reduce the complexity of atomic motion by transforming the dataset into a set of eigenvectors and eigenvalues. PCA was performed to extract dominant collective motions from the equilibrated trajectory segments. RMSD, RMSF, Rg, and PCA analyses were performed using GROMACS commands over the equilibrated portions of the MD trajectories [79]. In addition, ligand RMSD and binding-site Cα RMSD analyses were performed to evaluate ligand retention and local structural stability. Cluster analysis was conducted on the equilibrated trajectories to identify dominant conformational states and assess binding-mode persistence [80].

#### 4.8.2. Free Binding Energy Analysis

The free binding energy of the protein–ligand complexes was estimated using the MM-PBSA method based on the final 100 ns of the equilibrated trajectory. This analysis was conducted to evaluate the overall binding stability of each complex. Calculations were performed using the gmx_MMPBSA tool (v1.6.3) integrated with GROMACS. The binding free energy was calculated by considering contributions from van der Waals forces, electrostatic interactions, polar solvation energy, and nonpolar solvation energy [81].

#### 4.8.3. Free Energy Landscape and Dynamic Cross-Correlation Analyses

Understanding the energy landscape of the MD trajectories is essential for evaluating the internal motions and conformational stability of the protein–ligand complexes. Based on the principal components, the FEL was constructed by projecting the trajectory along the first two principal components (PC1 and PC2) and visualized using Origin2025. The FEL provides insights into the thermodynamically stable and metastable conformational states sampled during the simulation. Free energy values were calculated using the equation:

∆G=−kBTlnP

where kB is the Boltzmann constant, T is the absolute temperature (300 K), and P represents the probability density.

A DCCM analysis was performed in R (v4.2.2) using the Bio3D package (v2.4.5) R (Bio3D) with Cα atoms extracted from the equilibrated portions of the trajectories. This analysis reveals how different regions of the protein move in relation to each other, which can offer valuable insights into allosteric effects or cooperative behavior within the structure [82].

### 4.9. DFT-Based Quantum Chemistry Analysis 

To understand the molecular reactivity of the active compounds, DFT calculations were performed using Orca 6.1.0, and all calculations were conducted in the gas phase without implicit solvent correction [83]. Initial ligand geometries were built in Avogadro 1.2.0 and subsequently optimized at the B3LYP/def2-TZVP level with D3BJ dispersion correction. Vibrational frequency calculations were then performed at the same level of theory to verify the stability of the optimized structures. Single-point energy calculations were carried out on the optimized geometries to obtain frontier molecular orbital (HOMO and LUMO) energies, dipole moment, and Gibbs free energy. Additionally, MEP surfaces were generated and visualized using UCSF Chimera to analyze charge distribution and identify potential reactive regions on each ligand [84]. To further evaluate site-specific reactivity, Fukui indices were calculated using the finite difference approximation based on Hirshfeld population analysis. For each compound, three single-point energy calculations were performed at the same level of theory on the neutral (N electrons), anionic (N + 1 electrons), and cationic (N − 1 electrons) species. Hirshfeld atomic charges were extracted from each calculation and used to compute the condensed Fukui indices as follows:

$$f^{+}=q(N+1)-q(N);$$

$$f^{-}=q(N)-q(N-1);$$

$$f^{0}=(f^{+}+f^{-})/2$$

where q(N), q(N + 1), and q(N − 1) represent the Hirshfeld atomic charges of the neutral, anionic, and cationic species, respectively, and f^+^, f^−^, and f^0^ represent the susceptibility toward nucleophilic, electrophilic, and radical attacks, respectively. Atoms exhibiting the highest f^−^ values were considered the most susceptible to electrophilic attack and were compared with the hydrogen bond acceptor and hydrophobic interaction sites identified in the molecular docking analysis to evaluate the consistency between electronic reactivity and ligand–protein interaction patterns.

## 5. Conclusions

The present study identifies *C. sappan* as a promising source of structurally diverse phytochemicals that target complementary regulatory hubs within the shared AD–T2DM molecular network. By integrating systems biology, docking, MD simulations, MM-PBSA, and DFT analyses, the present study provides multi-scale computational evidence that structurally distinct phytochemical subclasses of *C. sappan* may coordinately modulate inflammation, kinase signaling, proteostasis, and cellular stress pathways. These findings support the potential of *C. sappan* phytochemicals as multi-target modulators and provide candidate molecules for subsequent experimental validation targeting interconnected neurodegenerative and metabolic disorders.

## Figures and Tables

**Table 1:** Classification, physicochemical, drug-likeness, and toxicity profiles of selected compounds from *C. sappan*.

No.	Compound Name	Classification	Physicochemical Properties					Pharmacokinetics and Drug-Likeness Properties			
			Molecular Weight (g/mol) (<500)	LogP (<5)	H-Bond Donors (≤5)	H-Bond Acceptors (≤10)	TPSA (<140)	BBB (0–0.3)	Lipinski Rule (Violation ≤ 1)	AMES Toxicity (Yes/No)	hERG Inhibitor I (Yes/No)
1	Sappanchalcone	Chalcone	286.28	2.47	3	5	86.99	0.05	Accepted	No	No
2	Quercetin	Flavonol	302.24	2.16	5	7	131.36	0.008	Accepted	No	No
3	Rhamnetin		316.26	2.88	4	7	120.36	0.005	Accepted	No	No
4	Tetraacetylbrazilin	Homoisoflavonoid	454.43	2.67	0	9	114.43	0.155	Accepted	No	No
5	Protosappanin B		304.29	1.60	5	6	110.38	0.045	Accepted	No	No
6	Sappanol		304.29	1.33	5	6	110.38	0.046	Accepted	No	No
7	Protosappanin C		302.28	1.38	4	6	107.22	0.012	Accepted	No	No
8	Sappanone B		302.28	1.77	4	6	107.22	0.041	Accepted	No	No
9	Brazilin		286.28	2.16	4	5	90.15	0.031	Accepted	No	No
10	Episappanol		304.29	1.22	5	6	110.38	0.029	Accepted	No	No
11	Caesalpin J	Phenol	316.31	1.22	3	6	96.22	0.065	Accepted	No	No
12	alpha-Amyrin	Pentacylic triterpenoid	426.72	7.60	1	1	20.23	0.056	Accepted	No	No
13	beta-Amyrin		426.72	7.71	1	1	20.23	0.081	Accepted	No	No
14	Lupeol		426.72	6.80	1	1	20.23	0.015	Accepted	No	No
15	Taraxerol		426.72	6.78	1	1	20.23	0.122	Accepted	No	No
16	Linolenic acid	Fatty acid	278.43	3.15	1	1	37.3	0.004	Accepted	No	No
17	Arachidic acid		312.53	8.39	1	1	37.3	0.017	Accepted	No	No
18	Linoleic acid		280.45	4.60	1	1	37.3	0.014	Accepted	No	No
19	Myristic acid		228.37	5.82	1	1	37.3	0.163	Accepted	No	No
20	Oleic acid		282.46	6.17	1	1	37.3	0.021	Accepted	No	No
21	Palmitic acid		256.42	6.73	1	1	37.3	0.06	Accepted	No	No
22	Stearic acid		284.48	7.57	1	1	37.3	0.029	Accepted	No	No
23	1-Octacosanol	Fatty alcohol	410.76	11.88	1	1	20.23	0.003	Accepted	No	No
24	beta-Sitosterol	Sterol	414.71	8.03	1	1	20.23	0.136	Accepted	No	No
25	Stigmasterol		412.69	7.50	1	1	20.23	0.178	Accepted	No	No
26	D-Galactose	Monosaccharide	180.16	−2.10	5	6	110.38	0.292	Accepted	No	No
27	D-erythro-Pentose, 2-deoxy-		134.13	−1.97	3	4	77.76	0.179	Accepted	No	No
28	Triacontane	Alkane	422.81	13.97	0	0	0	0.002	Accepted	No	No

**Table 2:** Molecular docking binding energies and interactions of protein and ligands from *C. sappan*.

Protein	Ligand	Binding Affinity (kcal/mol)	Interacting Residues	
			Hydrogen Bond	Others (Type)
SRC	Quercetin	−9.1	Met341, Glu339	Leu273, Leu393, Val281 (π-sigma), Ala293, Lys295, Ala403 (π-alkyl)
	Rhamnetin	−9.1	Met341, Glu339	Leu273, Leu393, Val281 (π-sigma), Ala403, Lys295, Ala293 (π-alkyl)
	Tetraacetylbrazilin	−8.7	Ser345, Lys295	Leu393 (π-sigma), Ala403, Val281, Leu273 (π-alkyl)
	Dasatinib (DB01254)	−9.2	Thr179, Glu178, Arg155	Cys185 (π-sulfur), Arg155, Lys203, Arg175 (π-cation)
	Native ligand (H8H)	−9.8	-	Ala403, Ala293, Lys295, Leu325, Val281, Leu393, Leu273, Tyr340 (alkyl), Val281, Leu273 (π-alkyl)
STAT3	Sappanone B	−7.7	Asp158, Glu62, Tyr27, Cys157	Ile22, Val30, Leu147 (π-alkyl), Tyr27 (π-π-stacked), Lys45 (π-cation)
	D-Galactose	−4.7	Glu24, Cys157, Gln96	-
	Protosappanin B	−8.4	Asp102	Leu147, Lys45, Val30, Ala43 (π-alkyl)
	Protosappanin C	−8.3	-	Lys45, Val30, Ala43, Leu147 (π-alkyl)
	Caesalpin J	−7.1	Gln96, Asp158	Lys45, Cys157 (π-alkyl)
	Celecoxib (DB00482)	−8.1	Lys105	Cys157, Ala43, Lys45, Val30 (alkyl), Leu147 (π-sigma)
	Native ligand (5ID)	−6.5	Asn145, Ser144, Lys105	Ile22, Ala43, Val30 (alkyl), Leu147 (π-sigma)
p53	Sappanchalcone	−7.8	His297, Glu356	Tyr335, Ile223 (π-alkyl), Ala226 (π-sigma)
	Myristic acid	−5.1	Asn265, Lys294	Ala226, Trp352 (alkyl), Ile223 (π-alkyl)
	PhiKan 083 (DB08363)	−7.5	His293, Tyr335	Ile223 (π-alkyl), Trp352 (π-π-stacked), Ala226 (π-sigma)
	Native ligand (SAH)	−7.2	Val277, Asn265, Gly264, Ala261, Tyr335, Ser225	Ala226 (π-alkyl), Trp352 (π-π-stacked)
HSP90α	Protosappanin B	−8.6	Trp162, Gly108	Leu107 (π-alkyl), Phe138 (π-π-stacked), Met98 (π-sulfur)
	D-Galactose	−5.2	Asp93	-
	D-erythro-Pentose, 2-deoxy-	−4.1	Tyr139, Trp162	-
	Geldanamycin (DB02424)	−8.2	Gly137, Phe138	-
	Native ligand (ONJ)	−10.3	Leu48	Ala111, Val186, Leu107 (π-alkyl), Ala55 (π-sigma), Met98 (π-sulfur), Asn51 (π-donor hydrogen bond), Tyr139 (π-π-T shaped)

**Table 3:** MM-PBSA binding free energy calculations from MD trajectories.

Protein–Ligand Complex	MM-PBSA (kcal/mol)						
	van der Waals	Electrostatic	Polar Solvation (EGB)	SASA Energy (ESURF)	GGAS	GSOLV	Total
SRC- quercetin	−41.42 ± 5.87	−12.01 ± 8.00	32.21 ± 6.25	−5.50 ± 0.53	−53.43 ± 8.19	26.71 ± 6.19	−26.72 ± 5.23
SRC- rhamnetin	−22.23 ± 4.30	−22.33 ± 12.54	30.46 ± 8.87	−3.55 ± 0.63	−44.56 ± 13.71	26.91 ± 8.48	−17.66 ± 6.47
SRC- tetraacetylbrazilin	−34.48 ± 2.89	−20.13 ± 8.75	37.11 ± 7.64	−4.96 ± 0.40	−54.60 ± 9.51	32.15 ± 7.41	−22.46 ± 3.79
SRC-dasatinib	−52.70 ± 4.18	−69.16 ± 11.40	105.25 ± 11.80	−7.26 ± 0.51	−121.86 ± 12.74	97.99 ± 11.53	−23.88 ± 3.85
STAT3- sappanone B	−26.66 ± 3.78	−36.49 ± 11.36	40.13 ± 7.11	−4.45 ± 0.57	−63.14 ± 12.86	35.67 ± 6.70	−27.47 ± 7.11
STAT3- protosappanin B	−30.42 ± 3.50	−44.39 ± 8.33	47.68 ± 6.69	−5.02 ± 0.29	−74.81 ± 7.43	42.65 ± 6.74	−32.15 ± 4.92
STAT3- protosappanin C	−17.41 ± 3.22	−49.58 ± 6.51	43.30 ± 4.18	−3.75 ± 0.19	−66.99 ± 5.92	39.54 ± 4.11	−27.45± 2.97
STAT3- celecoxib	−22.78 ± 3.29	43.77 ± 9.79	−16.73 ± 8.21	−3.36 ± 0.36	20.99 ± 9.23	−20.08 ± 8.16	0.90 ± 3.28
p53- sappanchalcone	−25.75 ± 3.65	−37.38 ± 8.09	42.33 ± 5.54	−3.30 ± 0.16	−63.13 ± 7.51	39.03 ± 5.48	−24.09 ± 3.94
p53- PhiKan 083	−25.33 ± 2.61	−333.29 ± 18.86	340.41 ± 18.32	−3.52 ± 0.29	−358.62 ± 19.25	336.90 ± 18.17	−21.72 ± 2.69
HSP90α- protosappanin B	−36.77 ± 0.76	−15.86 ± 3.47	37.56 ± 1.67	−3.48 ± 0.02	−52.63 ± 3.55	34.08 ± 1.67	−18.55 ± 3.92
HSP90α- geldanamycin	−39.41 ± 2.84	−15.72 ± 8.07	35.33 ± 6.68	−5.44 ± 0.35	−55.13 ± 8.27	29.89 ± 6.60	−25.24 ± 3.34

GGAS: van der Waals + electrostatic; GSOLV: EGB + ESURF; Total: GGAS + GSOLV.

**Table 4:** DFT-based thermodynamic, electronic, and reactivity parameters of *C. sappan* active compounds.

Ligand	Thermodynamic and Electronic Properties		Frontier Molecular Orbital (FMO)			Global Reactivity Descriptors				
	Gibbs Free Energy (kcal/mol)	Dipole Moment (Debye)	HOMO (eV)	LUMO (eV)	HOMO-LUMO Gap (eV)	Electronegativity, χ (eV)	Chemical Hardness, η (eV)	Chemical Potential, μ (eV)	Chemical Softness, S (eV)	Electrophilicity Index, ω (eV)
Quercetin	−691,897	5.5472	−5.8652	−1.9868	3.8784	3.9260	1.9392	−3.9260	0.2578	3.9742
Rhamnetin	−716,520	0.3098	−5.6378	−2.0269	3.6109	3.8324	1.8055	−3.8324	0.2769	4.0674
Tetraacetyl- brazilin	−1,005,552	4.0857	−6.1925	−0.9689	5.2236	3.5807	2.6118	−3.5807	0.1914	2.4545
Sappanone B	−670,103	5.5116	−5.7691	−1.8650	3.9041	3.8171	1.9521	−3.8171	0.2561	3.7319
Protosappanin B	−670,825	3.3955	−5.6444	−0.5687	5.0757	3.1066	2.5379	−3.1066	0.1970	1.9013
Protosappanin C	−622,939	3.7620	−5.7399	−1.3367	4.4032	3.5383	2.2016	−3.5383	0.2271	2.8433
Sappanchalcone	−271,152	5.0973	−5.9090	−2.0685	3.8405	3.9888	1.9203	−3.9888	0.2604	4.1427

HOMO: Highest occupied molecular orbital; LUMO: Lowest unoccupied molecular orbital.

## Data Availability

The original contributions presented in this study are included in the article/Supplementary Materials. Further inquiries can be directed to the corresponding author.

## Supplementary Materials

The following supporting information can be downloaded at https://www.mdpi.com/article/10.3390/ijms27125300/s1.

## Abbreviations

The following abbreviations are used in this manuscript:

AD	Alzheimer’s disease
T2DM	Type 2 diabetes mellitus
PPI	Protein–protein interaction
MNC	Maximum neighborhood component
EPC	Edge percolated component
*SRC*	SRC proto-oncogene, non-receptor tyrosine kinase
*STAT3*	Signal transducer and activator of transcription 3
*HSP90AA1*	Heat shock protein 90α
*TP53*	Tumor protein p53
GO	Gene Ontology
KEGG	Kyoto Encyclopedia of Genes and Genomes
MD	Molecular dynamics
RMSD	Root-mean-square deviation
RMSF	Root-mean-square fluctuation
Rg	Radius of gyration
PCA	Principal component analysis
MM-PBSA	Molecular Mechanics Poisson–Boltzmann Surface Area
FEL	Free energy landscape
DCCM	Dynamic cross-correlation matrix
DFT	Density functional theory
FMO	Frontier molecular orbital
HOMO	Highest occupied molecular orbital
LUMO	Lowest unoccupied molecular orbital
MEP	Molecular electrostatic potential
FDR	False discovery rate
PDB	Protein Data Bank
